# Entropy, Economics, and Criticality

**DOI:** 10.3390/e24020210

**Published:** 2022-01-28

**Authors:** Michael S. Harré

**Affiliations:** Complex Systems Research Group, Faculty of Engineering, The University of Sydney, Sydney 2006, Australia; michael.harre@sydney.edu.au

**Keywords:** criticality, phase transitions, network theory, economics, financial markets, non-linear dynamics, non-stationary processes, tipping points

## Abstract

Information theory is a well-established method for the study of many phenomena and more than 70 years after Claude Shannon first described it in A Mathematical Theory of Communication it has been extended well beyond Shannon’s initial vision. It is now an interdisciplinary tool that is used from ‘causal’ information flow to inferring complex computational processes and it is common to see it play an important role in fields as diverse as neuroscience, artificial intelligence, quantum mechanics, and astrophysics. In this article, I provide a selective review of a specific aspect of information theory that has received less attention than many of the others: as a tool for understanding, modelling, and detecting non-linear phenomena in finance and economics. Although some progress has been made in this area, it is still an under-developed area that I argue has considerable scope for further development.

## 1. Introduction

Information theory as a tool for economics has a long history and at this point is well-established in many sub-fields. This foundational work was carried out by authors such as Kolmogorov [1], Theil [2], Wilson [3], Georgescu-Roegen [4], and Aoki [5], each contributing significantly to quite different fields within economics. A recent review of information theory at the agent level and economics can be found here [6] and a broader review of entropy in economics in general over the last 150 years can be found here [7]. Recently, many others have contributed key results to this body of work. At the market level Vogel et al. [8] used data compression and information theory to study different regimes of a financial market. Sornette has also written an earlier but highly informative review of work on markets as exhibiting critical phenomena in their non-linear dynamics [9]. At a similar scale but for housing markets Crosato et al. [10,11] have studied the criticality of city dynamics using maximum entropy techniques. At the individual agent level Dinis et al. [12] studied phase transitions in optimal betting strategies using the Kelly criterion.

This opinion piece argues for an alternative use of information theory that has been used earlier but has yet to make a significant impact in the field of economics or information theory: As a tool for the analysis of “critical phenomena” in economics. It follows on from earlier work I have completed applying the notion of critical phenomena to the abrupt breaks in time series data, such as market crashes, such as the 1987 crash [13], the Asian crisis of 1997 [14], the build up to the housing crisis of 2007 [15], and the COVID-19 crisis of 2020–2021 [16], all of which made use of information theory in its various forms. With collaborators, I have also explored the occurrence of bifurcations in micro-economics [17,18], as well as in housing markets [19,20], each using maximum entropy techniques. In order to make the case for information theory as a tool in the analysis of criticality in economics, I will argue for two important elements. The first is that critical phenomena, i.e., bifurcations, catastrophes, tipping points, etc., can be analysed most effectively using information theory due its intrinsic sensitivity to non-linear behaviour. The second is that market dynamics exhibit behaviour that is very much like what we should expect in critical phenomena. These two points are covered in the next two sections and then some final points are discussed at the end.

## 2. Criticality and Statistical Measures

I begin by outlining and connecting some well established results that, to the best of my knowledge, have not previously been discussed in combination with each other. There is an early result due to Theil [21] that establishes the relationship between the amount of information shared between the dependent and the independent variables in a multiple regression analysis, and that it can be derived directly from correlations and partial correlations. The result is easy to state. Given a multiple regression analysis between a dependent variable $X_{0}$ and *N* independent variables $X_{i}\in \left\{X_{1},X_{2},\ldots X_{N}\right\}=X$, the question arises as to how much each $X_{i}$ contributes to the behaviour of $X_{0}$. Given the total correlation *R* between $X_{0}$ and X and the correlations $r_{0,1}$ between $X_{0}$ and $X_{1}$, partial correlations $r_{0,2|1}$ between $X_{0}$ and $X_{2}$ conditional on $X_{1}$, etc., then the total amount of information contributed from each of the $X_{i}$ to $X_{0}$ is a sum of their individual information contributions:

(1) $$I\left(R^{2}\right)\&=\&I\left(r_{0,1}^{2}\right)+I\left(r_{0,1|2}^{2}\right)+\ldots +I\left(r_{0,N|1,2,\ldots ,(N-1)}^{2}\right)$$

where $I\left(x\right)=-\log_{2}\left(1-x\right)$ and as x is bounded on the interval [0,1] this is a non-negative value corresponding to the information content, see the paper and references therein for the details. Contrast this approach to Scheffer et al. [22] (Box 3), in which the relationship between the non-stationary properties of the auto-correlation coefficient of an AR(1) process as it approaches a tipping point is analysed and we see that to first order in Equation (1), i.e., $I(r_{1,0}^{2})$, there is an informational analogue to Scheffer et al.’s analysis of the precursory signals of an impending tipping point.

There has already been progress in developing this direction as Barnett and colleagues [23] studied the relationship between “Granger causality” (G-causality), first developed by Granger in econometrics [24], and transfer entropy (TE). According to G-causality, given a vector X of stochastic variables that evolve in time it is said that “$X_{i}$ G-causes $X_{j}$” if, by including $X_{i}$ in the predictive information set of $X_{j}$, the subsequent prediction of $X_{j}$ is improved beyond the extent to which $X_{j}$ is already able to predict its own future. The central insight of the work of Barnett et al. is that, for Gaussian variables, G-causality is equivalent to TE, making a direct connection between the predictive analysis of vector auto-regression and information theoretical approaches to causal inference. This becomes relevant to critical phenomena not only because of the relationship with Scheffer et al.’s work but also because TE peaks before the phase-transition in the two-dimensional Ising model [25], where the Gaussian assumption no longer holds, i.e., TE becomes a candidate for the analysis of phase transitions at precisely the point where the relationship between TE and G-causality is expected to break down.

There is a similar correspondence between Pearson correlations and mutual information (MI) in the Ising model. Away from the critical temperature there is an exponential decay in the correlations between the individual spins. However, as the temperature approaches the critical temperature, the relationship between correlations and MI become strongly non-linear, although still expressible in closed form [26]. A distinguishing characteristic between MI and TE is that TE peaks before the phase transition (on the disordered side of the transition) whereas the MI peaks (diverges) exactly at the phase transition.

A final example is the use of Kullback–Leibler divergence (KL-divergence) to measure the statistical separation between probability distributions. Both MI and TE are specific examples of KL-divergence but the more general form is useful in its own right. It is defined for two discrete probability distributions P(X) and Q(X) for $X=\{X_{1},X_{2},\ldots ,X_{n}\}$ as:

(2) $$D_{KL}\left(P;Q\right)\&=\&\sum_{i}P\left(X_{i}\right)\log\left(\frac{P(X_{i})}{Q(X_{i})}\right)$$

Although the KL-divergence is central to MI and TE, it is also central to other information measures, specifically the Fisher information (FI) which has also been used in the study of critical phenomena. To see the relationship start with a θ-parameterised family of distributions P(X|θ), then the KL-divergence between two members of this family is $D_{KL}\left(\left(X|\theta \right);\left(X|\theta^{\prime }\right)\right)$ and as the divergence is minimised (zero) when $\theta =\theta^{\prime }$ we can expand this around θ to second order in $\theta^{\prime }$ [27] (Section 3.3: Information Measures):

(3) $$D_{KL}\left(P\left(X|\theta \right);P\left(X|\theta^{\prime }\right)\right)\&=\&\frac{1}{2}{\left(\theta^{\prime }-\theta \right)}^{T}\left(\frac{\partial^{2}}{\partial \theta_{i}^{\prime }\partial \theta_{j}^{\prime }}D_{KL}\left(P\left(X|\theta \right);P\left(X|\theta^{\prime }\right)\right)\right)\left(\theta^{\prime }-\theta \right)+h.o.t.$$

where the matrix of second derivatives (the elements are denoted $\partial_{i,j}D\left(\theta ;\theta^{\prime }\right)$) in this equation is the FI matrix, i.e., the FI is the first non-zero term in the expansion of the KL-divergence about $\theta =\theta^{\prime }$. We note that the FI is known to measure the gain in transient sensitivity of a distribution [28]. In that work, Prokopenko et al. were able to relate the $\partial_{i,j}D\left(\theta ;\theta^{\prime }\right)$ terms to the rate of change in the corresponding order parameters θ. Of relevance to the current article is that these relationships allow for the identification of second-order phase transitions via the divergence of individual $\partial_{i,j}D\left(\theta ;\theta^{\prime }\right)$ terms of the FI matrix. This work was later generalised to the Fisher TE [29], which was used to capture both transient and contextual aspects of the second order phase transition of the two-dimensional Ising model.

## 3. Critical Transitions Are a Phenomena of Markets

One of the first approaches to using statistical measures to understand sudden behavioural changes in financial markets is the work of Onella et al. [30] on the “Black Monday” crash on 19 October 1987. In that study they used a modified form of the Pearson correlation coefficient to measure the dyadic relationships between pairs of equities. This measure begins with the correlations between all pairs of equities $\rho_{i,j}$ and transforms them into a distance measure $d_{i,j}=\sqrt{2(1-\rho_{i,j})}$ which results in a distance matrix that can be thought of as a network of distances between equities. The underlying correlations were based on a window of time: $\left[t_{a},t_{b}\right]$ that was a subset of the complete time series, and then through a sliding of this window over the whole time series a sequence of equity trees could be built up and the dynamical properties of the market correlations could be studied as Black Monday approached. What was observed is that the equity tress collapsed to a star network at the point of the market crash, very similar to the abrupt transitions observed in the topologies of networked systems going through a phase transition [31]. This network approach has been applied in theoretical and empirical studies of networked equity markets in order to test the robustness of the phase transition idea, see for example the work of Kostanjvcar et al. [32].

This approach was extended to information theoretical measures in two distinct ways in order to further understand market crises as critical phenomena. In the first instance, the $d_{i,j}$ measures were replaced with MI and the same analysis as that of Onnella et al. was carried out for the subsequent MI network [15]. In that study, it was shown that there are peaks in the MI at crisis points as predicted by the Matsuda et al. [26] study of the Ising model, and, furthermore, the MI could be broken up into its entropy and joint entropy components in a diagnostically informative fashion, for example distinguishing between the market disruption of the 11 September 2001 attacks which had no discernible increase in joint entropies, only an increase in the entropy terms, and the 1987 crisis which had a significant increase in the joint entropies. It was also noted that there was a peak in MI away from any known critical points, suggesting that MI may be identifying other non-linear transients indicative of the market restructuring in more subtle ways than market crashes. In a second extension to the Onnela work a modified version of TE that accounts for the continuous flow of information through the market (rather than artificially discretising the data) was applied to the Asian financial crisis of 1997 [14] in order to build a network of information flows around a market crisis point. The key finding was that Pearson correlations and continuous TE distinguish between qualitatively distinct aspects and that continuous information flows are a more sensitive measure of dynamics during a crisis.

Other approaches to modelling criticality in economic markets have focused on the application of potential functions to market dynamics in order to test for the statistical significance of tipping points in uni-variate times series. In several recent papers [33,34,35], researchers have used the stochastic form of Thom’s catastrophe theory put forward by Cobb [36] and Wagenmakers et al. [37] to examine the empirical evidence for critical transitions in housing and equity markets. This follows on from a recent re-evaluation of catastrophe theory in economics as argued for in the review by Rosser, Jr. [38]. In principle, if a system has a well-defined potential function (sometimes “potential landscape”), a necessary element for catastrophe theory, then the system should also be susceptible to the methods proposed in a variety of fields [39,40,41,42] for the detection of nearby critical transitions, which brings us back to the study mentioned above by Scheffer et al. [22]: nearby critical points can (sometimes) be detected using statistical methods that measure the progressive deformation of the probability distributions caused by the deformation of the potential function near a critical point.

## 4. Limitations and Future Directions

The points laid out above are not without their issues and there are reasonable arguments for why this approach is less attractive than traditional statistical methods. However, I believe most of these can be addressed and here I divide them into two broad classes: issues of practice and issues of principle.

Some practical issues are the same for every discipline that uses information theory: the computations are expensive, there are fewer out-of-the-box software packages available, and more data are needed to obtain statistically reliable results. The first point is simply one that we may need to accept, even as computers become faster, it will likely remain the case that, for example, the Pearson correlation will be faster to compute than the corresponding MI. However, the computations will get faster in absolute terms as computers get faster, and other fields with large datasets, such as neuroscience, have seen the benefits of these new methods [43,44] with efficiency gains being made as well [45,46]. On the other hand software packages are becoming more readily available, and economics can benefit from the software advances that have been made in other fields. Two popular packages that have come from neuroscience are JIDT [47] and TRENTOOL [48] and their successor IDTxL [49]. As the methods become more readily used no doubt more implementations will become available. The data problem is a constant issue in economics, independent of the arguments made above, and aside from financial and industrial economics where data are rich and progress was initially quite rapid as computer scientists and physicists worked on market dynamics [50], data tends to be more sparse. However, while infrequently sampled time series and non-stationary data can make long term temporal analysis and prediction problematic, there is often considerable high resolution geospatial data, for example tax revenue or house prices indexed by postcodes. From this point of view long term prediction may still be difficult but temporally localised dynamics is achievable if the information theory tools can be adapted accordingly to suit the task at hand. It is also hoped that as data limitations more clearly become the bottleneck to better analyses, then private and government agencies will gather more data and this will become less problematic.

Another issue arises though, and it is a matter of principle rather than practice: Is information theory more useful than simply a new tool? In neuroscience and artificial intelligence there is a good “in principle” argument for why information theory is useful, it measures the amount of information being stored, processed, and transmitted within a complex adaptive system. For example Zhu et al. [51] studied neuromorphic nanowire networks using TE and active information storage, finding that information theoretical values peak when the networks transition from a quiescent state to an active state, illustrating the relationship between information theory as a measure of computational capacity and criticality in an artificial system. Likewise, other studies have shown that biological brains may be poised at or near a critical state [52] where it has been argued the brain is at a point of “self-organised criticality”, a term introduced by Bak [53], and see the recent critical review by Girardi-Schappo [54]. Others have argued that this may be a widespread property of many other systems as well, see, for example, the recent article by Tadić and Melnik [55]. However, the case has yet to be made that, at this more conceptual level, information theory and criticality adds to economic discourse, so I would like to discuss one, rather speculative, path through which this is relevant to economics. The point is fairly straightforward, Mirowski and Somefun [56] and Axtell [57], amongst others, have argued that markets and economies are computational processes in their own right, as Axtell frames it:

There is a close connection between agent computing in the positive social sciences and distributed computation in computer science, in which individual processors have heterogeneous information that they compute with and then communicate to other processors.

This is very much in the same vein as how neuroscientists might describe the processing of information in the human brain at the neuronal level [58]. The analogy does not map across in a trivial way though and care is needed. In a financial market, for example, instead of electrical signals between neurons price movements are the primary means of communicating and coordinating economic activity, and this might, at a suitably high level, justify the view that a market or an economy is indeed a computational process. However, market traders do not form long term price signalling relationships between each other in the way that neurons form connections with one another and so we need to be careful about the precise specification that comes from this analogy. One way in which we can keep the brain–market analogy but make it more pertinent is to take the recent work of Solé on “liquid brains” [59] that have been used as a computational model of ant communities as the analogy, rather than the “solid brains” of neural networks with their more rigid connections. This brings us to the point of self-organised criticality in economics and why it might be a relevant lens through which to see market dynamics. In this view, which has been espoused several times in recent work [60,61,62], markets need to be able to sensitively adapt to informational changes in such a way that allows prices to reflect news, and like liquid brains a critical or near critical state of the market may be the most effective position in order for a market to do so.

## Data Availability

No data was used in this article.
